# Peripheral Thyroid Hormone Sensitivity Mediates the Association Between Body Composition and Diabetes in Euthyroid Adults

**DOI:** 10.1002/jcsm.70061

**Published:** 2025-09-14

**Authors:** Nanfang Yao, Lan Liu, Yuqi Jiang, Dongmei Wang, Qianyi Lu, Yi Zeng, Genfeng Yu, Qintao Ma, Peiyi Li, Lingling Liu, Jie Shen, Heng Wan

**Affiliations:** ^1^ Department of Endocrinology and Metabolism The Eighth Affiliated Hospital of Southern Medical University (The First People's Hospital of Shunde) Foshan Guangdong China; ^2^ School of Nursing Southern Medical University Guangzhou Guangdong China; ^3^ Guangdong Engineering Technology Research Center of Metabolic Disorders Interdisciplinary Precision Prevention and Digital Healthcare The Eighth Affiliated Hospital of Southern Medical University (The First People's Hospital of Shunde) Foshan Guangdong China; ^4^ Department of Nutrition Shunde Hospital of Southern Medical University (The First People's Hospital of Shunde) Foshan Guangdong China

**Keywords:** appendicular skeletal muscle mass, body composition, diabetes, fat‐to‐muscle ratio, peripheral thyroid hormone sensitivity

## Abstract

**Background:**

Body composition parameters, including fat‐to‐muscle ratio (FMR) and appendicular skeletal muscle mass (ASM), are closely associated with metabolic diseases. However, the mediating role of peripheral thyroid hormone sensitivity, reflected by the free triiodothyronine‐to‐free thyroxine ratio (FT3/FT4 ratio), in these associations remains unclear in euthyroid individuals. The current study aimed to investigate the associations of FMR and ASM/BMI with diabetes and to explore the potential mediating role of FT3/FT4 ratio in these relationships among euthyroid Chinese adults.

**Methods:**

This cross‐sectional study enrolled the participants from 10 communities in Guangdong Province, China, from November 2021 to September 2022. Body composition was measured using bioelectrical impedance analysis (BIA). Diabetes was defined based on fasting plasma glucose, 2‐h postload glucose or glycated haemoglobin (HbA1c). Associations of FMR and ASM adjusted for body mass index (ASM/BMI) with diabetes were assessed using multivariable logistic regression. Structural equation modelling was applied to evaluate the mediating effect of FT3/FT4 ratio.

**Results:**

A total of 5089 participants (mean age = 46 years, SD = 12) were included in the final analysis, consisting of 1957 men (38.5%) and 3132 women (61.5%). Participants in the third tertile (T3) of FMR exhibited a 79% higher prevalence of diabetes compared to those in the first tertile (T1) (OR 1.79, 95% CI 1.27–2.54) (*p* < 0.05). Conversely, those in the T3 of ASM/BMI had a 56% lower prevalence of diabetes compared to participants in T1 (OR 0.44, 95% CI 0.31–0.64) (*p* < 0.05). Restricted cubic spline analysis confirmed linear associations for both FMR and ASM/BMI with diabetes (both *p* for overall < 0.001; *p* for non‐linear > 0.05). FT3/FT4 ratio significantly mediated the association between ASM/BMI and diabetes, accounting for 4.6% of the total effect (*p* < 0.05), whereas no significant mediation was observed in the FMR–diabetes pathway (*p* > 0.05).

**Conclusions:**

In euthyroid individuals, higher FMR and lower ASM/BMI were independently associated with increased diabetes prevalence. The inverse relationship between ASM/BMI and diabetes may be partially mediated by FT3/FT4 ratio, highlighting peripheral thyroid hormone sensitivity as a potential mechanistic biomarker linking ASM loss to glycaemic dysregulation in euthyroid individuals.

## 1 Introduction

Over the past three decades, the prevalence of diabetes has risen sharply, becoming a major global and national health concern [1]. In China, the prevalence is projected to reach 12.4% within the next decade, imposing significant health and economic burdens [2]. Consequently, there is a pressing need to identify modifiable risk factors and potential mediators early to enable timely interventions and optimized management strategies.

Skeletal muscle plays a central role in glucose metabolism, and muscle mass loss has been recognized as a key risk factor for diabetes [3]. Notably, reduced muscle mass can also pose metabolic risks in individuals with normal or low BMI, including younger adults—a population in which diabetes incidence is rising but often underrecognized [4, 5]. Appendicular skeletal muscle mass adjusted for BMI (ASM/BMI) has been identified as a superior index of muscle health and is recommended by the Asian Working Group for Sarcopenia [6, 7]. At the same time, individuals with similar BMI may present markedly different fat distributions, contributing to heterogeneous metabolic risks [8]. As such, the fat‐to‐muscle ratio (FMR) is increasingly recognized as a complementary indicator of body composition [9]. However, few studies have evaluated the associations of ASM/BMI and FMR with diabetes in Asian populations, particularly among Chinese adults.

Thyroid hormones are key regulators of metabolism [10]. Free triiodothyronine (FT3) and free thyroxine (FT4) are the physiologically active forms of thyroid hormones and are the genuine reactions of thyroid hormone metabolism [11]. The FT3/FT4 ratio reflects deiodinase activity and can be used to assess peripheral thyroid hormone sensitivity [12, 13]. Emerging evidence indicates that the conversion of free FT4 to the biologically active free FT3 predominantly occurs in peripheral tissues such as ASM [14, 15]. Loss of skeletal muscle mass can impair local deiodinase activity, disrupting the FT3/FT4 ratio [14, 15]. Moreover, thyroid hormones are increasingly recognized for their involvement in glucose metabolism, with FT3 influencing insulin signalling and glucose utilization in both hepatic and peripheral tissues [16, 17]. However, the potential effect that FT3/FT4 ratio mediates the associations between body composition and diabetes in euthyroid individuals remains unclear.

Therefore, this community‐based cross‐sectional study aimed to investigate the associations of body composition indices, including FMR and ASM/BMI, with diabetes and to explore the potential mediating role of FT3/FT4 ratio in these relationships among euthyroid Chinese adults.

## 2 Materials and Methods

### 2.1 Study Design and Subjects

This study used data from the Study on Prevalence of Metabolic Diseases and Risk Factors in Shunde (SPEED‐Shunde), an ongoing community‐based prospective cohort initiated in Shunde District, Foshan, Guangdong Province, South China. We performed the SPEED‐Shunde cohort, enrolling adult residents through multistage stratified cluster sampling across 10 communities. Baseline data were collected between November 2021 and September 2022. All investigators were trained using standardized protocols, and data collection was performed by a single clinical team from the Department of Endocrinology, Shunde Hospital of Southern Medical University. Detailed descriptions of the SPEED‐Shunde have been published previously [18]. The study was approved by the Ethics Committee of Shunde Hospital of Southern Medical University (Approval No. 20211103) following the Declaration of Helsinki. All participants provided written informed consent before participation.

**FIGURE 1:**
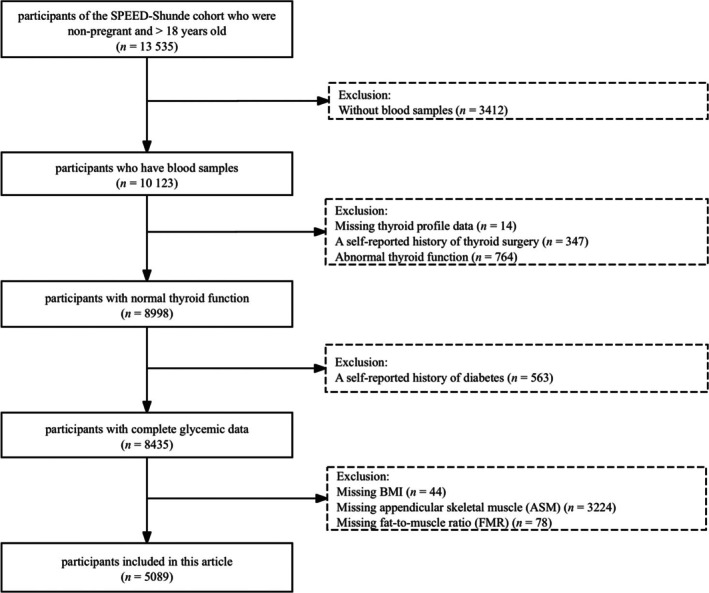
Flow chart of the study participants.

The inclusion criteria were as follows: (1) age ≥ 18 years and (2) local residency for ≥ 6 months. Participants were excluded if they (1) were pregnant, (2) had an acute illness or severe communication impairment or (3) refused to participate in the study. A total of 13 535 participants were initially enrolled in the SPEED‐Shunde cohort. We excluded participants who were without blood sample data (*n* = 3412), missing thyroid function data (*n* = 14), with a self‐reported history of thyroid surgery (*n* = 347), with abnormal thyroid function (*n* = 764), with a self‐reported history of diabetes (*n* = 563), missing BMI (*n* = 44), missing ASM (*n* = 3224) and missing FMR (*n* = 78). Finally, 5089 euthyroid participants with complete information were included in the final analysis (Figure 1).

### 2.2 Demographic, Behavioural and Anthropometric Assessment

Participants completed a standardized questionnaire to report sociodemographic information, including age, sex, education level, smoking status and alcohol consumption. Education was categorized into three levels: less than high school, completed high school and beyond high school. Smoking status was classified as current smokers (≥ 100 cigarettes smoked in a lifetime and smoking currently or within the past 6 months), former smokers (quit smoking for more than 6 months) and non‐smokers [19]. Alcohol consumption was reported as standard drinks per day and converted to grams (1 drink = 14 g of alcohol). Abusive drinking was defined as > 30 g/day for men and > 20 g/day for women [20]. Trained investigators followed a uniform protocol to measure height and weight. BMI was calculated as weight in kilograms divided by height in metres squared (kg/m^2^). Blood pressure was measured twice on the nondominant arm using an automated electronic sphygmomanometer (HEM‐752 FUZZY; Omron, China) after a 5‐min seated rest, with a minimum 1‐min interval between readings. The average values of systolic blood pressure (SBP) and diastolic blood pressure (DBP) were recorded. Hypertension was defined as SBP ≥ 140 mmHg or DBP ≥ 90 mmHg and/or a history of hypertension [21].

### 2.3 Laboratory Indicators

Participants fasted for at least 10 h overnight before fasting blood samples were collected between 7:00 am and 10:00 am. Serum levels of FT3, FT4 and thyroid‐stimulating hormone (TSH) were measured using a Microparticle Enzyme Immunoassay (MEIA) on the UniCel Dxi 800 Access System (Beckman Coulter, USA). The reference ranges were as follows: FT3, 3.53–7.37 pmol/L; FT4, 7.98–16.02 pmol/L; and TSH, 0.56–5.91 mIU/L [19, 20]. A standard 75‐g oral glucose tolerance test (OGTT) was administered to participants without a prior history of diabetes. Blood samples for fasting plasma glucose (FPG) and 2‐h postload plasma glucose (PPG) were collected in sodium fluoride anticoagulant tubes. Only FPG was measured among participants with self‐reported diabetes. Plasma glucose, total cholesterol (TC), triglycerides (TGs), low‐density lipoprotein cholesterol (LDL‐C) and high‐density lipoprotein cholesterol (HDL‐C) were analysed using an automated biochemical analyser (AU5831, Beckman Coulter, USA). HbA1c was assessed using high‐performance liquid chromatography (HLC‐723G8; TOSOH, Japan). Dyslipidaemia was defined as meeting any of the following criteria: TC ≥ 6.22 mmol/L, TG ≥ 2.26 mmol/L, LDL‐C ≥ 4.14 mmol/L, HDL‐C < 1.04 mmol/L or current use of lipid‐lowering medications [22]. All blood samples were transported under cold chain conditions to a central laboratory certified by the College of American Pathologists, where they were centrifuged and stored at −20°C within 2 h of collection.

### 2.4 The Definition of Diabetes

Diabetes was defined according to established diagnostic criteria recommended by the American Diabetes Association (ADA) [23]. A diagnosis of diabetes was assigned if any one of the following conditions was met: (1) glycated haemoglobin (HbA1c) level ≥ 6.5%, (2) FPG level ≥ 7.0 mmol/L or (3) 2‐h PPG level ≥ 11.1 mmol/L following a standard OGTT [23, 24].

### 2.5 The Definition of ASM/BMI and FMR

Body composition parameters, including body fat, total muscle mass and ASM, were measured using a multi‐frequency bioelectrical impedance analysis (BIA) device (InBody770 machine, Seoul, South Korea) [18]. Participants were instructed to avoid strenuous exercise, alcohol intake and heavy meals for at least 12 h before the measurement. All assessments were performed in the morning after an overnight fast. During the measurement, participants stood barefoot on the footplate electrodes and held the hand electrodes with both hands while maintaining a relaxed, upright posture, ensuring full contact with all electrodes. ASM was calculated as the sum of lean soft tissue mass in both arms and legs. ASM was adjusted for body size using the following formula: ASM (kg)/BMI (kg/m^2^) [6, 25]. FMR was defined as total fat mass (kg) divided by total muscle mass (kg) [26, 27].

### 2.6 Statistical Analysis

Baseline characteristics were presented as mean (standard deviation [SD]) or median (interquartile range [IQR]) for continuous variables and as number (percentage) for categorical variables. The Kolmogorov–Smirnov test was used to assess the normality of distributions. Differences between groups were compared using the independent samples *t*‐test, Mann–Whitney *U* test or chi‐squared test, as appropriate.

Multivariable logistic regression models were applied to assess the associations of ASM/BMI and FMR with the prevalence of diabetes. Both indices were categorized into tertiles, with the lowest tertile (T1) as the reference. Models evaluating ASM/BMI were adjusted for age, sex, education level, smoking status, alcohol consumption and hypertension. Analyses involving FMR included additional adjustments for BMI. Subgroup analyses were conducted by BMI (< 24 and ≥ 24 kg/m^2^), age (< 50 and ≥ 50 years) and sex. To further assess the non‐linear relationships, restricted cubic spline (RCS) regression was conducted with 3–5 knots placed at prespecified percentiles (5th, 25th, 50th, 75th and 95th). Variance inflation factor (VIF) was calculated to evaluate multicollinearity, and all covariates included had VIF values < 5.

Structural equation modelling (SEM) was conducted to examine whether the FT3/FT4 ratio mediated the association between body composition and diabetes. ASM/BMI and FMR were treated as independent variables, FT3/FT4 ratio as the mediator and diabetes as the outcome. Model fit was assessed using the comparative fit index (CFI), Tucker–Lewis index (TLI) and root mean square error of approximation (RMSEA). Standardized path coefficients were used to interpret the mediation effect.

All statistical analyses were performed using SPSS Version 23.0 (IBM Corporation, Armonk, NY, USA) and R software Version 4.3.0 (R Foundation for Statistical Computing, Vienna, Austria). A two‐tailed *p* value < 0.05 was considered statistically significant.

## 3 Results

### 3.1 Characteristics of Participants

The characteristics of the study population are presented in Table 1. A total of 5089 participants (mean age 46 years, SD 12) were included, comprising 1957 (38.5%) men and 3132 (61.5%) women. The prevalence of diabetes was 10.2%. Compared with participants without diabetes, those with diabetes were older and had higher BMI, HbA1c, FPG, PPG, FT3 and FMR values (all *p* < 0.001). The diabetes group also exhibited a higher prevalence of hypertension (36.5% vs. 20.5%) and dyslipidaemia (50.9% vs. 31.6%). The FT3/FT4 ratio and ASM/BMI were significantly higher in the diabetes group (*p* < 0.05). Additionally, educational attainment was lower, and the proportion of current smokers and abusive drinkers was slightly higher in the diabetes group.

**TABLE 1:** Baseline characteristics of the study population.

	Overall participants		
	Without diabetes	With diabetes	*p*
*N*	4568	521	
Age	45.67 (11.79)	53.17 (11.31)	< 0.001
Sex			0.040
Male	1735 (38.0%)	222 (42.6%)	
Female	2833 (62.0%)	299 (57.4%)	
BMI	23.53 (3.35)	25.03 (3.66)	< 0.001
HbA1c	5.58 (0.40)	6.55 (1.29)	< 0.001
FPG	4.58 (0.58)	6.26 (2.25)	< 0.001
PPG	7.16 (1.64)	14.04 (3.57)	< 0.001
FT3	5.33 (0.57)	5.44 (0.57)	< 0.001
FT4	11.27 (1.42)	11.30 (1.45)	0.605
FT3/FT4 ratio	0.48 (0.07)	0.49 (0.08)	0.015
FMR	0.41 (0.14)	0.46 (0.15)	< 0.001
ASM	18.04 (4.30)	18.44 (4.49)	0.067
ASM/BMI	0.77 (0.16)	0.74 (0.16)	< 0.001
Hypertension			< 0.001
No	3630 (79.5%)	331 (63.5%)	
Yes	938 (20.5%)	190 (36.5%)	
Dyslipidaemia			< 0.001
No	3126 (68.4%)	256 (49.1%)	
Yes	1442 (31.6%)	265 (50.9%)	
Education			< 0.001
Less than high school	1606 (35.2%)	252 (48.4%)	
Completed high school	1112 (24.3%)	138 (26.5%)	
Beyond high school	1850 (40.5%)	131 (25.1%)	
Smoking			0.043
Non‐smoker	3964 (86.8%)	438 (84.1%)	
Former smoker	138 (3.0%)	12 (2.3%)	
Present smoker	466 (10.2%)	71 (13.6%)	
Abused drink			0.043
No	4446 (97.3%)	499 (95.8%)	
Yes	122 (2.7%)	22 (4.2%)	

*Note:* Participants' baseline characteristics were summarized as mean (standard deviation [SD]) for continuous variables and as numbers (percentages) for categorical variables.

Abbreviations: ASM, appendicular skeletal muscle mass; BMI, body mass index; FMR, fat‐to‐muscle ratio; FPG, fasting plasma glucose; FT3, free triiodothyronine; FT4, free thyroxine; HbA1c, glycated haemoglobin; PPG, postload plasma glucose.

### 3.2 Associations of FMR and ASM/BMI Tertiles With Diabetes

**FIGURE 2:**
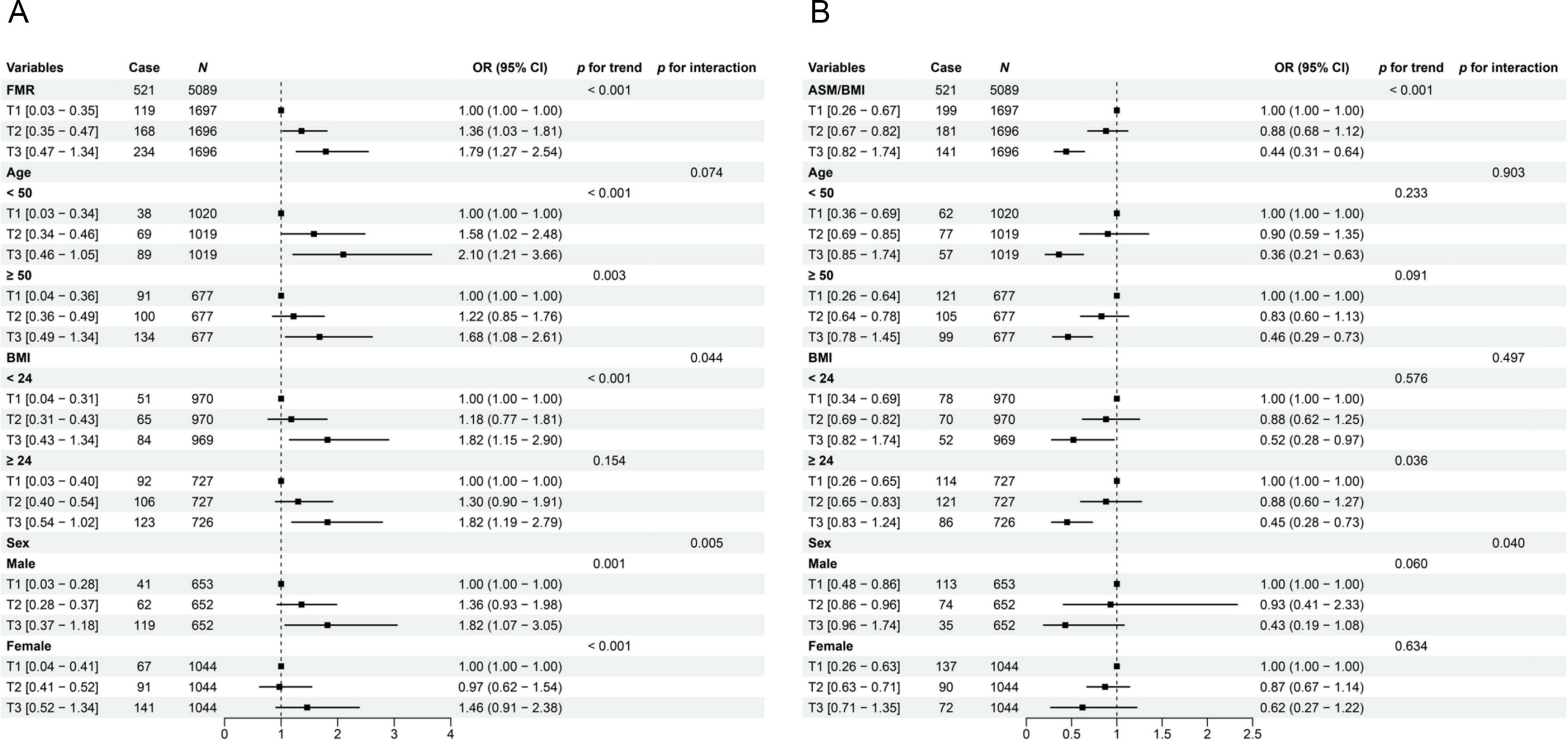
Associations between body composition indices and diabetes across subgroups. Odds ratios (ORs) for (A) fat‐to‐muscle ratio (FMR) and (B) appendicular skeletal muscle mass (ASM)/body mass index (BMI) were estimated using logistic regression models. Models evaluating ASM/BMI were adjusted for age, sex, education level, smoking status, alcohol consumption and hypertension. Analyses involving FMR included additional adjustments for BMI.

Figure 2 shows the associations between FMR and ASM/BMI tertiles and the prevalence of diabetes across subgroups. Compared with T1, the ORs (95% CI) for diabetes across increasing FMR tertiles (T2–T3) in the overall population were 1.36 (1.03–1.81) and 1.79 (1.27–2.54) (*p* for trend < 0.05). Subgroup analyses showed consistent positive associations in age, BMI and sex strata. Interestingly, compared with men among the T1, those among FMR T3 had an 82% higher prevalence of diabetes (OR 1.82, 95% CI 1.07–3.05) (*p* = 0.030); however, this significant association was not observed among women (*p* > 0.120) (*p* for interaction = 0.005) (Figure 2A).

In contrast, ASM/BMI was inversely associated with diabetes. Compared with T1, the ORs (95% CI) across increasing ASM/BMI tertiles were 0.88 (0.68–1.12) and 0.44 (0.31–0.64) (*p* for trend < 0.05). Subgroup analyses also showed inverse associations in age, BMI and sex strata. Interestingly, compared with men among the T1, those among ASM/BMI T3 had a 57% lower prevalence of diabetes marginally (OR 0.43, 95% CI 0.19–1.08) (*p* = 0.056); however, this significant association was not observed among women (*p* = 0.210) (*p* for interaction = 0.04) (Figure 2B).

### 3.3 Non‐Linear Associations of FMR and ASM/BMI With Diabetes

**FIGURE 3:**
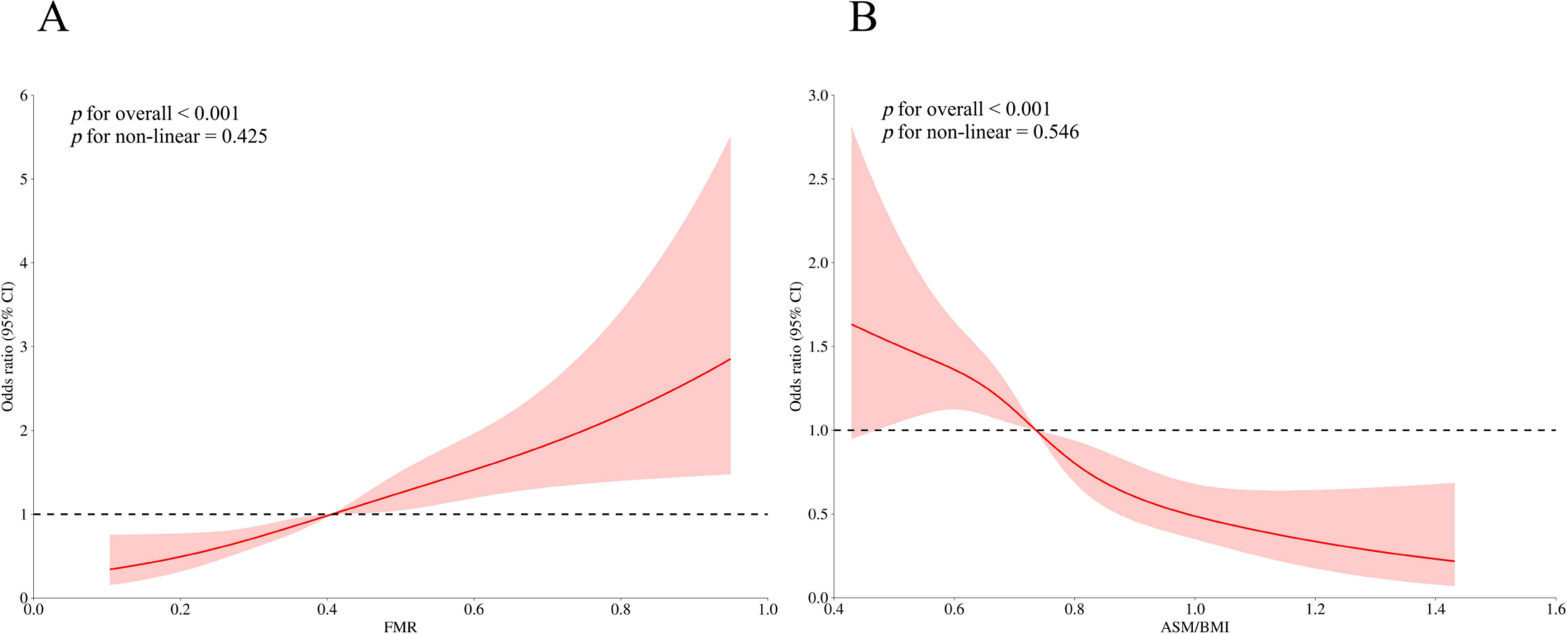
Restricted cubic spline analysis for the association between body composition indices and diabetes. Spline curves represent odds ratios of (A) fat‐to‐muscle ratio (FMR) and (B) appendicular skeletal muscle mass (ASM)/body mass index (BMI) with diabetes risk. Models evaluating ASM/BMI were adjusted for age, sex, education level, smoking status, alcohol consumption and hypertension. Analyses involving FMR included additional adjustments for BMI.

We utilized RCS models to examine the non‐linear associations between body composition indices and the prevalence of diabetes. As shown in Figure 3, FMR exhibited a significant positive association with diabetes prevalence (*p* for overall < 0.001), with no evidence of non‐linearity (*p* for non‐linear = 0.425) (Figure 3A). In contrast, ASM/BMI showed a significant inverse association with diabetes prevalence (*p* for overall < 0.001), also without significant non‐linearity (*p* for non‐linear = 0.546) (Figure 3B).

### 3.4 Mediation Analyses

**FIGURE 4:**
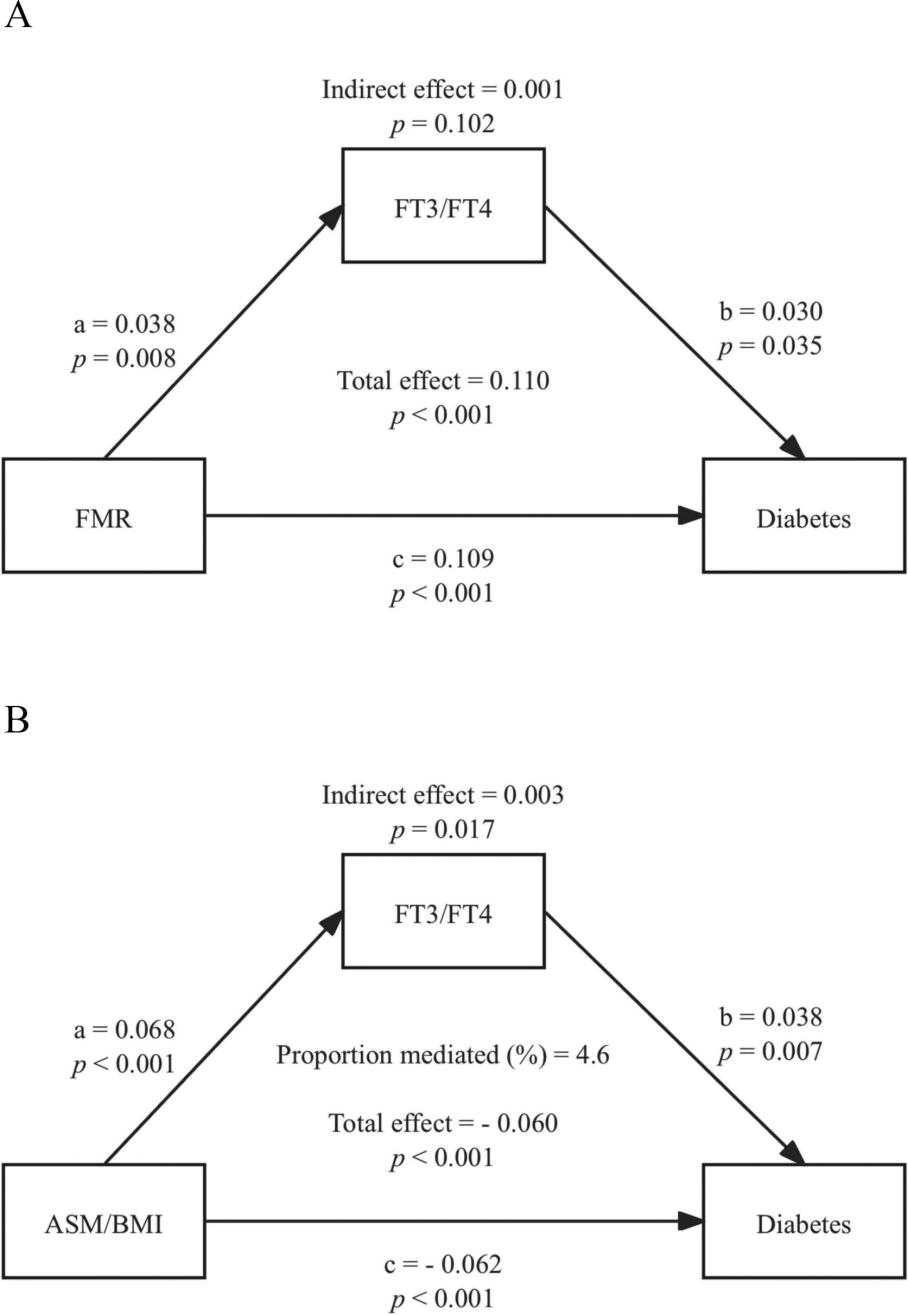
Mediation models assessing the role of free triiodothyronine‐to‐free thyroxine ratio (FT3/FT4 ratio) in the associations of fat‐to‐muscle ratio (FMR) and appendicular skeletal muscle mass adjusted for body mass index (ASM/BMI) with diabetes. Path ‘a’ represents the association between (A) FMR or (B) ASM/BMI and FT3/FT4 ratio, path ‘b’ represents the association between FT3/FT4 ratio and diabetes, and path ‘c’ represents the direct effect (DE) of FMR or ASM/BMI on diabetes after adjusting for FT3/FT4 ratio. Indirect effect (IE) = a × b; total effect = c + (a × b); proportion mediated = IE/(IE + DE). Models evaluating ASM/BMI were adjusted for age, sex, education level, smoking status, alcohol consumption and hypertension. Analyses involving FMR included additional adjustments for BMI.

As shown in Figure 4, FT3/FT4 ratio was evaluated as a potential mediator in the association of FMR and ASM/BMI with diabetes. No significant mediation was observed in the association between FMR and diabetes (indirect effect = 0.001, 95% CI 0.000–0.002, *p* = 0.102), although both the direct effect (*β* = 0.109) and total effect (*β* = 0.110) remained significant (*p* < 0.001) (Figure 4A). In contrast, the estimated indirect effect was 0.003 (95% CI 0.000–0.005, *p* = 0.017), accounting for approximately 4.6% of the total association (total effect = −0.060, *p* < 0.001), indicating a statistically significant partial mediation (Figure 4B). These findings suggest that FT3/FT4 ratio may serve as a partial mediator in the ASM/BMI–diabetes pathway but not in the FMR–diabetes pathway.

## 4 Discussion

In the current study, we found that FMR was significantly associated with an increased prevalence of diabetes, whereas higher ASM/BMI showed an inverse association. Notably, the FT3/FT4 ratio significantly mediated the association between ASM/BMI and diabetes but not between FMR and diabetes. These findings suggest that peripheral thyroid hormone sensitivity may partially explain the appendicular skeletal muscle–diabetes link among euthyroid adults.

The inverse association between ASM/BMI and diabetes observed in our study is consistent with findings from previous investigations [28]. For example, a cross‐sectional survey including 1388 Latin American adults also found a significantly negative association between ASM/BMI and diabetes [29]. In addition, reduced ASM/BMI has been associated with significant liver fibrosis in individuals with non‐alcoholic fatty liver disease [25]. A higher muscle mass, as defined by the ASM/BMI index, was associated with a lower risk of postpartum prediabetes and T2DM in Korean women with GDM [30] and cardiometabolic disorders [31, 32]. All the evidence suggests that the ASM/BMI index may be an essential risk factor for metabolic diseases.

The positive association between FMR and diabetes observed in our study is consistent with previous epidemiological findings. Several population‐based studies have reported that higher FMR is significantly associated with an increased risk of diabetes [27, 33]. As an integrated body composition index, FMR reflects the combined burden of excessive adiposity and reduced muscle mass, which are independently linked to metabolic disturbances [34, 35]. In addition, higher FMR values have been associated with metabolic syndrome and other cardiometabolic risks [36, 37]. These findings support the notion that unfavourable body composition, characterized by a disproportionate accumulation of fat relative to muscle, plays a crucial role in developing dysglycaemia and cardiometabolic risks.

The inverse association between ASM/BMI and diabetes observed in our study appears to be partially mediated by peripheral thyroid hormone sensitivity, as reflected by the FT3/FT4 ratio. Skeletal muscle, particularly the appendicular muscles of the limbs, is a primary peripheral site for converting T4 to T3 through the activity of deiodinase type 2 enzymes [15, 38]. Therefore, loss of ASM could impair local T3 generation, leading to lower FT3/FT4 ratios and subsequent disturbances in glucose metabolism [39]. This pathway may explain why FT3/FT4 ratio mediated the association between ASM/BMI and diabetes in our analysis. In contrast, no significant mediation effect of FT3/FT4 ratio was observed in the association between FMR and diabetes. One plausible explanation is that FMR, as a composite measure of fat and total muscle mass, may not specifically capture the status of appendicular skeletal muscle where deiodinase‐mediated T4‐to‐T3 conversion predominantly occurs.

Skeletal muscle plays a central role in maintaining glucose homeostasis through insulin‐stimulated glucose uptake. At the molecular level, skeletal muscle expresses high levels of glucose transporter type 4 (GLUT4), critical for facilitating glucose entry into cells in response to insulin signalling [40]. Loss of skeletal muscle mass reduces the overall capacity for glucose disposal, contributing to systemic insulin resistance [41]. Furthermore, skeletal muscle is a major site for mitochondrial oxidative metabolism [42]. Impaired mitochondrial function in muscle tissue, often accompanying muscle loss, leads to reduced oxidative capacity, increased lipid accumulation and further disruption of insulin signalling pathways [42, 43]. These molecular alterations collectively contribute to the development of glucose intolerance and increase the risk of type 2 diabetes.

There are some strengths to the present study, such as the concurrent evaluation of FMR and ASM/BMI with diabetes and the integration of peripheral thyroid hormone sensitivity into the analysis framework. However, several limitations should be acknowledged. First, because of the cross‐sectional design, causal relationships between body composition, thyroid hormone sensitivity and diabetes cannot be determined. Animal studies are required to investigate the causal relationships and the role of peripheral thyroid hormone sensitivity. Second, the study population consisted solely of Han Chinese participants, which may limit the generalizability of the findings. Third, body composition was assessed by BIA rather than dual‐energy X‐ray absorptiometry, which may introduce measurement bias. Fourth, we could not identify the associations between body composition and the specific types of diabetes in the current study because we did not test for type 1 diabetes‐related antibodies. However, we speculate that most of the participants with diabetes in our cohort are likely to have type 2 diabetes, as the prevalence of type 1 diabetes is low. Further research is needed to explore the associations between body composition and various types of diabetes.

## 5 Conclusions

In euthyroid Chinese adults, higher FMR and lower ASM/BMI were independently associated with an increased prevalence of diabetes. Notably, peripheral thyroid hormone sensitivity, reflected by the FT3/FT4 ratio, partially mediated the inverse association between ASM/BMI and diabetes but not the FMR–diabetes association. These findings highlight the importance of evaluating body composition beyond BMI and suggest that peripheral thyroid hormone sensitivity may be a mechanistic biomarker linking ASM loss to glycaemic dysregulation in euthyroid individuals.

## Disclosure

The authors have nothing to report.

## Ethics Statement

Ethical approval was obtained from the Ethics Committee of Shunde Hospital of Southern Medical University.

## Consent

All participants signed an informed consent.

## Conflicts of Interest

The authors declare no conflicts of interest.

## Data Availability

The findings of this study are supported by data that may be obtained upon request from the corresponding author. The data are not publicly accessible because of ethical or privacy constraints.
